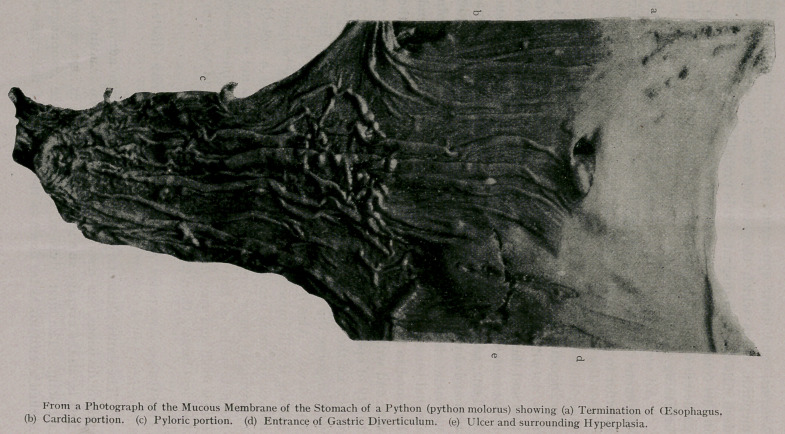# Some Original Investigations upon the Python Molurus

**Published:** 1890-08

**Authors:** J. Leffingwell Hatch

**Affiliations:** Lecturer on Bacteriology, and Assistant Demonstrator of Pathology in the University of Pennsylvania; Assistant Pathologist to Philadelphia Hospital, etc.


THE JOURNAL
OF
COMPARATIVE MEDICINE AND
VETERINARY ARCHIVES.

VOL. XI. AUGUST, 1890. No. 8.

### SOME ORIGINAL INVESTIGATIONS UPON THE PYTHON MOLURUS.

BY J. LEFFINGWELL HATCH, B.S., M.D.,
*Lecturer on Bacteriology, and Assistant Demonstrator of Pathology in the University of Pennsylvania; Assistant Pathologist to Philadelphia Hospital, etc.*

THE visceral anatomy of the pythonidæ, as far as its ultimate cellular nature is concerned, has not been thoroughly studied, or if it has been, was not described in any of the more prominent scientific works of either Europe or America. I have made a careful search of the literature from the works of Aristotle and Pliny up to the memoirs of the present day, and in none of them is the histology of the parts under consideration described. My main object in this paper will be to point out the probable meaning of a diverticulum of the stomach upon histological and morphological reasons; yet, for the sake of clearness in my line of thought and for the sake of comparison with other forms, I shall describe the histology of both the œsophagus and the stomach.

As it was through the meditation of a pathological inquiry that these studies were made possible, I shall devote a short space to a description of the lesion observed.

The pathology of this animal, even like the histology, has been sadly neglected. Comparative pathology has as yet done very little to help the showman, and those who have large collections of wild animals, to understand and judiciously treat the affections to which such animals are prone. It is true that veterinary medicine has made great advances and thrown much light on the diagnosis and treatment of diseases in the domestic animals, and thus saved much live stock that would otherwise have perished, but outside of the horse, cow, pig, sheep and dog, it has not as yet ventured. It is also true that M. Louis Pasteur, that great good man of France whose praises cannot be sung too often, has saved much money for the French Government and nation by teaching them the exact nature of the disease of the silk worm, of chicken cholera, and how to treat them; but just how far down in the animal kingdom we can trace pathological processes we know not.

It is a patent fact that the tendency to recovery from disease or the healing of a wound is the innate property of the organism to reproduce itself by cell multiplication when jostled from a state of stable equilibrium or fractured in the continuity of structure.

It is a fact well known to naturalists that the crab, one of the class of crustaceans, if it receives an injury to a portion of its limbs promptly breaks the limb off at a point higher up. This is accomplished by means of the extensor muscles, which pull the limb forcibly against the carapace, thus fracturing it at that point. The distal cells are then thrown into a state of proliferation by means of an active hyperæmia induced by the traumatism, and soon an entire new member is reproduced on the same plan and after the old pattern. The crab also undergoes a true ecdysis every year, throwing off the epithelial membrane of its alimentary tract from oral cavity to anus, save for the stomach, which preserves it in its pristine condition, so that here a pathological process due to cell outwandering and proliferation would be next to impossible, for after it was once nicely established it would be thrown off in toto and leave the crab with a clean new membrane throughout its entire tract.

Is it not then a difficult task to say just where the line between the possibilities of pathological processes or the harboring of "materies pecans" and conditions of complete exemption by means of a more perfect cell activity or individual assertion is to be drawn? To find out in just what forms of life complete immunity from these morbid changes exist and exactly to what their evolution is due, is a study that ought to go hand in hand with that of the "mechano-physiological philosophy of biology," which endeavors to point out the mechanical forces which brought about those changes that have adapted the organism to best survive in the struggle for existence.

The life of a cell depends upon eight fundamental principles, and it is the sum total of these which resist death, according to Bichat. Now, if disease is imperfect organization in imperfect action, the imperfect action must be due to the way in which some one or more of these fundamental conditions play their role. Accordingly, if we would understand the phenomena of perverted organic action, we must go to the ultimate source of trouble, the relation of the eight fundamental principles to the cell, and they are represented by its environment. As the lower forms of life are made up of fewer cells than their more cumbersome and complex relatives, the life history of the individual cell can be best studied among them, so that it seems to me that comparative pathology, or a science of disease that is based upon a knowledge of the different phases of a cell unit from the lowest forms up, is to be a science that will not only enlighten the student in biology but will aid the practical clinician of medicine.

In accordance with the above exposition I shall treat of ophidian life in general, the pythonidæ in particular, and study the anatomy and histology of the cells forming the parts diseased before endeavoring to reconcile the effect to any probable cause.

### History of Ophiology.

The first descriptions of serpents that we find anywhere in literature are those left to us in the precious works of Aristotle, but they are so marred by the prejudices and superstitious ideas of his time that they are worthless as an accurate description of the species that he chanced upon, and he speaks in a vague way of the aspic, the viper and serpents in general.

Pliny has also many curious stories to tell us about serpents, but as in his history generally, he has distorted the truth to please and occupy the reader with strange things, likewise in his accounts of snakes his elastic imagination has not disdained to stretch the truth. Nevertheless, he is the first to mention the principal species known at the epoch when he lived.

Then we have Ælianus following close upon Pliny, whose errors in description only exceeded those of Pliny by their number.

The other relics of classic ophiology were left to us by such writers as Nicander, Virgile, Lucian, etc., who speak more or less directly about serpents in their works.

The Greeks comprehended indifferently all serpents under the two names, δράχων and ὀΦὶς, which were derived from the verbs δέρχειν and ὄπτειν, both meaning to see. The German "schlange" comes from the verb schlingen, to turn or wind, and is analogous to the Latin serpens, from *serpere*, from which comes the French serpent that has been brought directly into English.

The word snake is of different etymology. It comes from A. S., "*snaca*," a snake (perhaps originally snáca).+Icel., *snákr*, *snókr;* Dan., *snog;* Swed., *snok.*+Skt., *nága*, a snake. Orig., "a creeper;" allied to the word sneak, which can be traced back through the Icel., Swed. and Dan. to the Gael. and Irish *snaig*, *snaigh*, to creep.

### Concerning Deglutition in Serpents.

Deglutition in the ophidians especially the pythonidæ, is a long and tedious process. They catch their prey alive and, if of the constrictor species, squeeze it into a pliable mass. They then force the animal far back into their throat, which is facilitated by the manner in which the lower jaw is articulated with the upper, a long narrow bone called the quadrate intervening between the two maxillæ; this allows the under jaw to open until it is parallel with the neck. The sharp recurved teeth keep the animal from slipping out, then the lower jaw advances while the upper holds on firmly, and thus by a play of the jaws, particularly the lower one, accompanied by a muscular effort known as swallowing, the mass is moved along the very long œsophagus, which is lubricated by a slimy mucus thrown out by numerous glands along its entire extent. In this manner small pigs, rabbits and birds are disposed of with comparative ease, and during not a very great length of time. Sometimes, however, a serpent will attempt to swallow too large an animal, and a portion will remain protruding from the mouth. Serpents found in this condition are very hideous, and their appearance, taken in connection with the mephitic odors which they exhale from the already decaying flesh, fills one with disgust. It is probably from ophidians found in this condition that material for the numerous fables has been gathered, a material which the poet has not been slothful in embellishing.

All serpents eat in the manner described above, save that the venomous ones hide their fangs in their sheaths during this act in order to protect them from injury.

### The Digestion of Ophidians.

Digestion among the ophidians takes place with great slowness, notwithstanding the activity of the stomach during digestion and the strength of the gastric juice. This fluid, however, is not secreted in great abundance, and many authorities, such as M. Lenz, supported by Schlegel, are of the opinion that maceration and chymification take place only at the pyloric end of the stomach. They bring forward as proof of this that on opening an animal some time after feeding the portion in the pyloric end will be found to have undergone great changes, while that at the cardiac end will scarcely be altered. I have never had the opportunity of opening a snake in this condition, so necessarily cannot vouch for the above statement. During captivity the ophidians will disgorge the undigested parts, such as feathers, hair, nails and claws. Also if one happens upon a snake that has just been feeding it will immediately empty itself that it may be more alert and escape more readily from enemies. This is a notable fact occurring in many birds. The digestive tract is not overtaxed in the pythonidæ, as they are dormant during the Winter season and refuse food, and even during the warmer season of the year they feed less largely than the carnivora.

### The Gross Anatomy and Description of the Œsophagus and Stomach.

The entire alimentary tract in the pythonidæ admits of two grand divisions, an œsophago-gastrial and an intestinal. The first is, in the average size adult python, 2.75 m., the second 1.75 m. long. The first division, composed of the œsophagus and stomach, extends as far down as the position of the right supra-

From a Photograph of the Mucous Membrane of the Stomach of a Python (python molorus) showing (a) Termination of Œsophagus. (b) Cardiac portion. (c) Pyloric portion. (d) Entrance of Gastric Diverticulum. (e) Ulcer and surrounding Hyperplasia.

renal capsule. The second division, joined to the first by a short transverse canal, the pylorus, is composed of the small and large intestines, which terminate in a cloaca on the under side of the body about 20 cm. from the end of the tail.

The œsophagus is quite capacious, having a circumference of 30 cm. throughout its entire length, and admitting easily the closed fist. It is highly elastic and expansible, is quite thin in structure, and translucent. In the recent condition it is collapsed on account of its property of elasticity. As it approaches the stomach it gradually becomes thicker and more opaque, on account of the increase of muscular fibres in the media. Its general course is in the median line and posterior to the trachea, to which it is adherent by loose connective tissues, but at a point in about the region of the heart it is deflected a little to the left and occupies a space between the left lung and the costal parieties ; this is evidently to favor respiration while deglutition is taking place, as aeration of the blood depends more upon the right lung than the left, the former being the larger and reinforced by an extensive air chamber at its extremity, while the latter is very small and often atrophied. The mucous lining throughout its entire length is smooth and presents a peculiar reticulated appearance. Numerous glands can be seen microscopically that empty upon its surface and keep it moist with a glairy mucous secretion, evidently for the purpose of aiding the passage of a large bolus of food.

Provision against completely shutting off the supply of air from pressure on the trachea is made by means of small muscles attached to the trachea, which not only enable it to be moved from side to side, but allow it to be projected from the mouth during the act of deglutition. There is scarcely any difference in size between the lower end of the œsophagus and the cardiac end of the stomach, the one gradually blending into the other, the shade of color and the tendency to the formation of minute longitudinal rugæ together with the increasing thickness pointing out the transition. (Vide photograph.)

The stomach rather than being a dilatation of the gut, as in many ophidians, is rather a continuance of the œsophagus for part of its course (so far as the measurement of its diameter is concerned, though in structure there is a great difference), then a marked narrowing takes place before it passes into the pylorus.

It is an elongated affair, having a total length of 1.50 m. and a diameter of 30 cm. at its widest part. It admits of division into two distinct parts in accordance with its form and structure. The first division commences at the cardiac orifice and extends for a distance of 1 m., the second is represented by that portion of the stomach between the point of termination of this first part and the pylorus. The first portion is musculo-membranous, and contains many longitudinal rugæ, which are entirely effaced when the stomach is greatly distended. The second part is narrow and thicker, the increase in thickness being due to an increase or super-addition of muscular fibres that from here on run in a longitudinal direction. The rugæ in the mucosa are less numerous, but are thicker and cannot be effaced by distention. The thickness of the walls, the size and number of the rugæ, together with the contraction of its circumference go to point out a definite line of separation between the two parts, which M. Duvernoy has proposed to call respectively "sac stomacal" (gastric sac) and "bayau pylorique" (pyloric bay).[1]

This division can readily be seen in the picture (vide plate), and when I come to speak of the histology of the stomach there are other differences to be observed that further aid us in the separation of the stomach into two portions.

On the outside of the stomach in the first portion, near the cardiac end, there is an oval body about the size of the gall bladder in man. It is a cyst or diverticulum of the stomach, into which it opens by means of a narrow duct. The walls are quite thick, and the cavity contains some sebaceous matter. The mucous surface corresponds exactly with that of the stomach, as does also its cellular elements, as will be seen in the chapter devoted to the histology of the stomach. The point of opening may be seen in the plate. This diverticulum, according to C. Poelman, is not always to be found in the same location, but may occur at the end of the "sac stomacal."[2]

It was considered by some of the earlier writers as the spleen,

[1] Fragments d'Anatomie sur l'organization des serpents. Memoire lu á l'Academie des Sciences de Paris, le 18 Juin, 1832, Annales des Sciences Naturalles de Paris, t. XXX, p. 135.

[2] Vide mem. cour. et mem. d. Sav. Etrong. de l'Acad. de Belg., Tom. 22, 1848, p. 5. Vide also J. P, Hopkinson and J. Pancoast. On the Visceral Anatomy of the Python, described by Daudin as the boa reticulata. Mit 1 taf. In Trans. Amer. Philos. Soc., N. S. vol. 5, 1837, pp. 121-134.

as that organ was for a long time overlooked, but Schlegel has pointed out the existence of a spleen in close proximity to the pancreas, with which organ it is intimately adherent.[1] Other writers have considered it either as anomalous or pathological.[2] No one has as yet pointed out its true nature, although M. Duvernoy, in the same article in which he comes to the conclusion that it is anomalous, speaks of it as a sort of rudimentary second stomach. This I believe to be the true nature of it, and in the chapter devoted to the discussion of its probable meaning I shall endeavor to prove it upon morphological and histological grounds.

### The Histology of the Œsophagus and Stomach.

I also made careful microscopical studies on the python which came under my observation. The results are briefly as follows:

The œsophagus consists of four coats, which, enumerated from without in, are as follows, viz: First, a fibrous covering which is eminently white fibrous elastic connective tissue; second, a muscular coat; third, an alveolar connective tissue, and fourth, a lining mucous membrane.

The muscular coat is quite regularly arranged, and is composed of striated muscular fibres. There are two layers of these muscles, one, the outer, running in a longitudinal direction, the other, inner, encircling the tube at right angles to the outer. The mucosa is made up of stratified epithelium, which rests upon a basement membrane and is encroached upon by numerous papillæ from the corium below. These papillæ are very close together, and under a high power show themselves to be made up of a core of areolar connective tissue in which courses bloodvessels, the whole being covered with columnar epithelium. The corium is separated from the rest of the alveolar coat by a thin layer of plain muscular fibres, the so-called muscularis mucosæ. The alveolar coat proper is between this and the internal muscular coat, and contains the larger branches of the bloodvessels and lymphatics, and also the mucous glands of the membrane, which are very large and which probably play a great part in the act of deglutition.

[1] Vide Essái sur la Physiomonie des Serpens, par H. Schlegel-La Haye, 1837, vol. I, p. 43.

[2] Fragments l'Anatomie sur l'organization des serpents. Memoire lu à l'Academie des Sciences de Paris, le 18 Juin, 1832, Annales des Sciences Naturalles, t. XXX, p, 133, par G. L. Duvernoy.

The above description holds good for the entire tract ; at the point where the œsophagus merges into the stomach, however, the coats become thicker, and the papillæ are gradually evolutionized into gastric glands.

The stomach, even like the œsophagus, has four coats, and they may be respectively called, following an order from the exterior to the interior, serous, muscular, areolar or submucous, and mucous membrane.

The serous coat is derived from the peritoneum and is endothelial in composition.

The muscular coat is divisible into three layers, a longitudinal, transverse and oblique.

The areolar or submucous coat serves to unite the mucous membrane with the muscular coat ; it is very loose in texture and in it ramify the larger branches of the bloodvessels and the lymphatics.

The mucous membrane is very thick and thrown into large longitudinal rugæ. The thickness is due to long tubular glands which empty into the interior by means of ducts. The papillæ between these glands is made up of areolar tissue containing capillaries. The mucous membrane is bounded below by the muscularis mucosæ, which consists of an external longitudinal and an internal circular layer of plain muscular fibres. The glands consist of a basement membrane lined with columnar epithelium. Those glands nearest the pylorus in the second portion of the stomach have larger cells, and also superadded parietal cells, the so-called oxyntic cells of Langley. It is these latter cells that secrete the powerful gastric juice of the ophidian stomach.

The bloodvessels reach the mucous membrane in the stomach in the usual way, along its curvatures, piercing the muscular coats and ramifying in the areolar structure. The capillaries penetrate clear up to the mucous membrane, passing between the glands in the papillæ ; at this point the arterioles terminate in a plexus of minute veins which encircle the mouths of the glands. These veins give rise to straight venous radicals, which pass back through the muscularis mucosæ and terminate in efferent veins, which leave the stomach in company with the efferent artery.

The lymphatics occur in t he mucous membrane in the form of a plexus or stomata ; these gaping mouths take the lymph, which is carried by means of larger valved vessels in the submucous coat, and efferent vessels which pass through the muscular coat to reach the serous membrane, beneath which they leave the organ.

The nerves are poorly represented, yet they occur in fine nonmedulated fibres, which form a plexus situated between the muscular coats, and giving off fine branches to the submucous coat.

The gastric diverticulum, described in the previous chapter, has the same structure as the stomach, as may be seen from the cuts.

The mucosa consists of tubular glands; these glands, however, do not possess parietal cells. The columnar cells on the borders of the papillæ are also smaller and less granular, probably from want of use.

### The Probable Meaning of the Gastric Diverticulum.

There have been a great many theories expressed about this pouch, from that of its being anomalous or pathological, to that of its taking the place of the spleen, but no one has analyzed it histologically before this time or I am sure it would have been better understood. As early as 1833 C. Poelman pointed out its existence, and as at that time the spleen had been overlooked he thought, from gross morphological appearances, that this must be that organ, and so described it in speaking both of the stomach and the spleen.[1]

He mentions the fact of its communicating with the stomach by means of a narrow orifice, that the walls are thick and that it contains a small quantity of sebaceous matter. The locality of this sac seems to be variable, for Poelman found it at the pyloric end, Hopkinson and Pancoast found it existing at both ends at the same time, while Schlegel, Duvernoy, Dujardine and Brown have found it constant, in numerous species examined, at the cardiac end, a place which it occupies in the python which I dissected. Poelman is held out in his views by Duvernoy, Hopkinson and Pancoast, the latter two gentlemen claiming two spleens for the reptile. Schlegel, however, in 1837, described some lobular bodies in connection with the pancreas, which he believed

[1] Vide Mem. Cour. et mem L. Sav. Etraug. de l'Acad. de Belg., Tom. 22, 1848, p. 5 et 11.

to be the spleen, and since that time his results have been verified by W. Müller, who worked up its histology in 1865 and described it in his work entitled "Ueber den feineren Bau der Milz."

In the latest work which we have on ophidians, *i. e.*, Bronn's "Klassen ŭnd Ordnung des Thiers-Reichs," we read that the spleen is usually bound more or less intimately to the anterior portion of the pancreas.[1]

I think from the foregoing exposition we are justified in concluding that this diverticulum does not play the role of a spleen, as that organ exists intact elsewhere; that it is not anomalous nor pathological seems patent from the fact of its invariable occurrence in every animal examined. Having thus knocked down the prop from the older hypotheses, what have I to offer in their place? In structure the walls are identical with those of the stomach; they have a serous, a muscular, an alveolar and a mucous coat, and the mucous membrane is made up of glands identical with gastric glands, save that there are no parietal cells.

From its morphology, size and histology, I am confident that this diverticulum is the remnant of a rumen, or, in other words, that it is a rudimentary second stomach. It probably plays a very unimportant role in the ophidian digestion, which would account for its atrophied condition.

### The Pathology of the Pythonidæ.

In the early part of February, this year, I was asked to examine a python that died while in confinement at the Zoological Garden in this city. From the symptoms before death Mr. Brown, the superintendent, concluded there was some gastric trouble, but of exactly what nature he was uncertain.

A systematic description of the morbid anatomy of the diseases to which these animals are prone seems to be veiled in the shadows of uncertainty and doubt. I have made an exhaustive search through the following works without finding a single paper upon the subject: *Index Medicus*, Virchow's *Archives*, *Zoological Record*, *Zoologischer Jahresbericht* (Berlin), *Zoologischer Anzeiger* (Leipsic), *Bibliotheca Zoologica* (Berlin), *Bibliotheca Zoologica* (Leipsic), *Verzeichniss der Schriften über Zoologie*.

In a small handbook entitled "An Introduction to General

[1] Vide Bronn's "Klassen ŭnd Ordnŭng des Thiers-Reichs," vol. I., S., 1883.

Pathology," by John Bland Sutton, F.R.C.S., however, I found a short account of a case of cancer in the stomach of the python, and as the python I posted was thought to have cancer of the stomach, before the autopsy, I shall give the case in full for the sake of comparison. (Compare p. 319 of the above-quoted book.)

"A python (python sebæ) which had lived in the Zoological Society's garden for fifteen years was found to be ailing, and as it grew worse it was deemed advisable to kill it. On making the post-mortem examination the viscera was found to be the seat of an enormous number of secondary growths. The liver measured three feet in length, and was studded with hard, yellowish-white nodules, varying in size from a pea to that of a large walnut. The lung contained twenty similar nodules of the size of peas. The kidneys had each a mass of new growths at their posterior ends, of the dimensions of a walnut. The ovaries had several deposits of the size of an orange, some being rather smaller. The nodules in all the organs were of a yellowish-white color, exceedingly hard to the touch, and many on being cut into exuded a greenish colored fluid. This was most obvious in the ovarian masses. In histological details they conformed to medullary cancer, being made up of alveoli containing masses of irregular cells. The alveolar walls are exceedingly thin, and in places difficult to distinguish. None of the growths were vascular, and the larger masses showed cavities, the result of disintegration of the morbid growths.

"It was impossible to decide as to the original seat of the growth, but, taking into consideration the size of the ovarian tumors and the relation of the blood stream to the other organs, it seems probable that the ovaries were the starting place of the mischief.

"The specimens are preserved in the museum of the Royal College of Surgeons, and figures of the cancerous nodules are given in the "Journal of Anatomy and Physiology," vol. xix."

From what I am able to gather from those who have the care of such animals, it seems that they invariably die of some gastro-intestinal trouble. Whether it is due to the effects of confinement or not can only be decided when we shall have had opportunity to study the lesions of those that die in their natural habitat and in a free state. This, it seems to me, could readily be done by those living in the regions where the python abounds.

The species, as we have seen, are limited and are not widely distributed, and if well worked up their pathology would be of great economic value. The question of mal-nutrition at once suggests itself to us in connection with disturbances of the alimentary tract, but the habits of the python have been so closely noted by many acute observers, that a mistake in the choice of pabulum is practically out of the question. If it be bacteridian, then here is a chance for the bacteriologist to spread himself and not only work out the cause, but search as well for a cure.

I found a number of parasitic worms in the pyloric end of the stomach in the python which I examined. That this is not an unusual occurrence I am confident, for Schlegel says that he has often found the pyloric end of the stomach completely filled with worms, which caused continued obstruction, and he believes were often the cause of death.[1]

These parasites encroach upon the walls of the stomach, and are sufficient cause to set up an inflammatory process.

The following genera have been met with in the stomach of the various species of ophidians : Ascaris, distoma, filaria, echinorhynchus, tænia, strongylus, trichosoma, pentastoma and cucullanus. For this information I am indebted to M. Rudolph.[2]

### The Clinical History of the Case.

In the snakes, as in the reptilia generally, the nervous system is of such a low grade that their sensations must obviously be very obtuse ; hence any painful process might exist without their manifesting any inconvenience from it, and suppuration go on almost to the entire destruction of a part without anything unusual being observed in the life habit of the animal. From this it will be seen how difficult it must necessarily be to elicit symptoms and make a diagnosis before a process of disease shall have advanced so far as to render interference useless.

For the few facts in the clinical history of the case under our consideration I am indebted to Mr. Brown, the superintendent of the Zoological Garden of this city.

[1] Vide Essai sur la Physionomie des Serpens, par H. Schlegel-La Haye, 1837, vol. I, p. 83.

[2] Vide Entozoorum, synopsis, p. 762.

About twelve months or so ago he noticed that the mucous membrane of the mouth showed foci of inflammation, these centres soon spread in a radiating manner and attacked the gums around the teeth, where the inflammatory process was followed by a caseous degeneration. There was also an excessive secretion of mucus, which the snake would blow sometimes from the mouth, then again from the nasal cavities; mucus was also passed per anum. There was marked anorexia even during the season when the python feeds best.

The snake was otherwise in good condition up to the time of death, and no other symptoms could be elicited. I have since seen the consort of the python that died, and it is evidently suffering from the same malady, if any reliance is to be placed upon the inflammatory process in the mouth as indicating a further ulcerative condition of the stomach.

How long this process is under way before it manifests itself thus it is hard to say, but all pythons that die in captivity, and all boas, if they live long enough before they die, are found to have this condition of the stomach.

The python I examined was probably over 100 years old. Reptiles grow very slow, and those of this species reach an enormous size. There are reports of pythons thirty feet long, and Pliny tells us that a python 120 feet long was killed on the shore of the river Bagrada by the soldiers in the army of Regulus during the Punic War. This was probably a species of the sub-family *Pythonicus holodontes*, and if 100 feet were taken off from the figures given a nearer approach to its exact length would be reached.

The largest snake I think we have any authentic record of was one sent to this country from Holland a few years ago, and measured a few inches over eighteen feet. A python eleven feet in length grows so slowly that in a period of two years from the time of measurement an increase in length is not perceptible. It takes an alligator from seventy-five to one hundred years to reach its full size and complete development, and as the rate of growth in the two animals is comparable, it certainly will take as long, if not longer, for a python to reach complete development as it does an alligator.

Some of the other colubers are prone to this affection. Mr. Brown says he has seen it in our common black snake.

I intend to carry on some experiments with this latter snake, and watch carefully the life history of this affection from as early a stage as possible, and thus try and throw some light on its clinical history, which is comparatively overshadowed with ignorance.

### The Post-Mortem—Some Details of My Case.

In order to preserve the skin for the museum, it was carefully removed, a process which took two days, so it was about three days after death before I could examine the organs.

The body was that of a well-nourished python (species molurus), 4.30 m. in length and 50 cm. around its thickest portion. The skin was covered with large imbricated scales, made up of a chitinous-like substance. The ventral portion was of a yellowish hue, the dorsal surface being mottled gray and black, following a fanciful design.

Around the pillars of the soft palate, at the side of the isthmus of the fauces, was a collection of mucus. I made some cultures from this on agar-agar and on gelatin, and obtained some growths which under the microscope proved to be micrococci. There was no inflammatory process in the mouth nor in the œsophagus. The lungs were crepitant and contained no diseased foci either of a primary or secondary nature. The heart was normal, having two auricles and one ventricle, with an incomplete septum. The liver was firm, of a dark-red color, and about 1 m. in length. It also showed no signs of a secondary deposit of any kind. The kidneys, as in all the lower forms of life, were lobulated, and, as is usual in ophidians, were long and narrow.

The supra-renal capsules were quite a long distance from them, about 20 cm. The ovaries and uterus were in good condition, opening into the cloaca in common with the intestine. The intestine was also free from disease. The stomach was the main point of interest. From the exterior, even before I had opened it, I could feel a hard mass as large as a small orange, and an external ulcer was also apparent on the serous surface directly over this mass. On opening the stomach it, like the rest of the alimentary tract, was found to be entirely devoid of food, the only matter present being a little mucus and a few parasitic worms.

The mucous membrane was quite moist and covered with a glairy and viscid mucus, through which the reticulated structure of the tissue could be seen with facility. The stomach measured 48 cm. in length and was 35 cm. at its broadest part.

The hard nodule that had been felt from without was situated at the cardiac end, and nearer the small rather than the large curvature. It was oval in shape, and considerably elevated above the surface of the stomach. It measured 14 cm. in length, 8 cm. in width, and was 2 cm. thick. There was a crater-like ulcer, 10.5 cm. in length, running along its greatest axis. There also appears in the picture a fissure at right angles to this, but this is merely an incision that I made in order to obtain sections to study microscopically, and close scrutiny will reveal the stitches where I sewed it together before photographing.

The free surface of the ulcer showed little granules that looked like tubercles; they were not tubercles, however, as I proved afterwards by my microscopical studies.

### The Histological and Bacteriological Studies Upon the Growth.

The neoplasm was firm to the touch and cut much like lymphoid tissue. I immediately made frozen sections and stained them with Bismarck brown and rosin. From these sections I was enabled to decide that it was not *cancer*, and that the bulk of its substance was lymphoid in nature.

The free surface of the ulceration showed little granules upon it that resembled in no small degree tubercles. Thinking that a specific granulomata might have to do with the growth, I made a culture from one of these on acid agar-agar, and obtained a luxuriant growth in a few days. I made cover-glass preparations from these and found them to be micrococci, 2.5$\mu$ in diameter. And I may here state that they corresponded, both in form and measurement, with those cultivated from the mucus found in the mouth.

I put the stomach in alcohol, and after it had become sufficiently hard made sections. Those from the different regions of the stomach have already been described, so I will now merely add the histology of the neoplasm.

I embedded them in celoidin, and cut them in the usual manner after such embedding. I stained some with carmine, others with hæmatoxalyn, and still others after the manner of Gram for bacteria.

I found the new growth to be made up of lymph cells, fibrous tissue and some mucous tissue. The lymphoid cells seemed to preponderate over the other elements, the fibrous tissue acting merely as a stroma. It appeared quite vascular, and resembled in a high degree granulation tissue.

The lymph spaces, as well as some of the capillaries, contained micrococci, which were identical with and measured the same as those described above.

### The Probable Nature of the Growth and Its Cause.

From the foregoing description of the histology of the growth it will at once be seen that we have here to do with an ulcerative process, and from the hyperplasia that it has been of long standing.

That it is probably septic the micro-organisms go to show, since they are found in the lymphatics and bloodvessels, as well as in the free surface of the growth.

Just how, where and when these bacteria entered the organism, and from whence they came, would be hard to predict. They are not evidently the forms of putrefaction or suppuration, being larger and having a different individual arrangement.

As has before been hinted, the kind of food could play but a very small role in the cause of a process like the above. It is true that the python swallows its food alive, or almost before it is dead, still a rabbit or a rat would be so paralyzed by the great pressure it must needs submit to before deglutition that it could do very little injury after it reached the stomach, even if it were not smothered to death during the long and tedious process of swallowing, as it probably is.

I have it from good authority, however, that sparrows have been heard to chirp after being for some time in the stomach of a boa. The feathers and claws and the undigested portions are as a rule vomited, thus showing that they act as foreign bodies there, stimulating the stomach to this reflex act. Very large bodies, however, I do not think could be cast up on account of the teeth, which are set in such a manner as to prevent the regurgitation of food while swallowing.

A boa constrictor has been known to swallow a blanket through sheer necessity, it having become entangled in the mouth of the boa. The teeth held it so firmly it could not be spit up, and the only way out of the difficulty was to swallow it.[1] The blanket was passed from the bowel some three weeks later in a cylindrical mass that tapered at each end.

Hæmorrhagic infarction is one of the great sources of ulcer in man. We have first a conical area of pigmentation due to a precipitation of hæmosiderine from the blood; this is acted upon by the gastric juice of the stomach, which causes a gradual erosion, and finally we get an ulcer, which, in accordance with the length of the process, may or may not have extended clear through the wall of the stomach, and has sharp and defined edges, as though it had been punched out with a conductor's punch.

In this form of ulceration, however, we do not meet with a bacterium, which puts this as a theory of cause beyond the pale of possibility.

The worms found in the stomach probably caused a great deal of irritation, and though they were found "en masse" at the pyloric end, nevertheless they could have originated the inflammatory process that went on to ulceration. And it seems to me a more rational idea to consider them as the exciting cause rather than accept without further proof a hypothesis of general cause.

To settle this, two experiments can be performed, viz: Give a python suffering with the earliest symptoms a vermifuge, and watch the results. If the inflammation is due to the presence of the worms it will be alleviated by their removal. Then, if this does not suffice, a germicide may be given, such as powdered naphthaline dusted over the feathers of a bird, or can be given by means of a tube passed into the stomach. The circulation of reptiles is too sluggish to serve the purpose of a hypodermic injection.

[1] For the authenticity of this I am dependent upon no less an authority than Joseph Leidy, M.D., Professor of Comparative Anatomy and Zoology in the University of Pennsylvania.

Mr. Brown and I propose experimenting in this direction at the Zoological Gardens, and it will give us great pleasure to publish the results of our work at the earliest possible moment.

The evidence of the present case goes to show that the cause lies between these two factors, and it remains but to eliminate one of these factors from our equation to find the unknown quantity.

### Bibliography.

*Blainville, H. de.*—Observations sur plusieurs Serpents du genre Python vivants á Paris, in Bull. Science, Soc. Philom., 1823, pp. 49–53. Tom. 96, 1823, pp. 271–277. Fror Nat. Bd., 5, No. 91, 1823, pp. 33–38.

*Cleyer, Andr.*—De Serpente magno Indicæ orientalis Urobubalum dedlutiente (Mit. 1 Taf.), in Ephemer. Acad. Nat. Cur., Dec. 2, Ann. 2, 1683 (1698), pp. 18–24.

*Duvernoy.*—Fragments D'anatomie sur l'organization des serpents. Memoire Lu á l'Académie des sciences de Paris, le 18 Juin, 1832, Annales des sciences naturelles de Paris, t. xxx, p. 135.

*Duges.*—Recherges sur lu déglutition des serpents. Ann. 8, Sc. Nat., 1827, xii, p. 362 et suiv.

*Gand.*—Memoire sur la structure et les fonctions de lu rate, 1846.

*Hopkinson, J. P., and Pancoast, J.*—On the visceral anatomy of the python, described by Daudin as the boa reticulata (Mit. 1 Taf.), in Transact. Amer. Philos. Soc. N. S., vol. 5, 1837, pp. 121–134.

*Jacquart, Henri.*—Memoire sur les organes de la circulation chez le Serpent Python (Mit. 3 Taf.), in Amer. Science nat., 4, Sér Zool. Tom. 4, 1855, pp. 321–364. De l'appareil circulatoire sanguin chez le Serpent Python in Compt. rend. Acad. Sc., Paris, Tom. 42, 1856, pp. 1125–1128.

*Meckel.*—Vergleichenden Anatomie. Zwei Bänd., Leipzig, 1808. Traite général D'anatomie comparée, Paris, 1838, t. viii, p. 73.

*Poelman, C.*—Note sur l'organisation de quelques parties de l'appareil digestif du python bivittatus (14 pag. Mit. 2, Taf.) in Mem. cour. et Mém. 8 Sav. Etrang. de l'Acad. de Belg., tom. 22, 1848. Ansz. Fror. Nat. 3, Réche, Bd. 6, No. 113, 1848, p. 74.

*Schlegel, H.*—Essai sur lu physionomie des serpents, La Haye, 1857, partie generale, p. 40.

*Sutton, John Bland, F.R.C.S.*—An Introduction to General Pathology, p. 319.

### Appendix.

Since completing this article I have received two pythons from the Zoological Garden which, during life, gave evidence of gastro-intestinal irritation. The serpents belong to the species

Regis, and were of the following dimensions: The larger of the two 56¾ inches in length, and 7½ inches around its largest girth. It weighed 4 pounds 10.5 ounces. The other was 55¼ inches long, 5¾ inches around its greatest girth, and weighed 2 pounds 12.5 ounces. They were both prettily marked and had just shed their skins, so that they appeared in good condition.

In the larger snake I found the following pathological conditions: The pylorus was intussuscepted within the stomach for a distance of two and one-half inches, and was withdrawn with considerable difficulty, springing back to its original position as soon as traction was discontinued. On opening the stomach the lesser curvature was found to be the seat of an ulcerated hyperplasia, giving much the same picture as that in the species molorus, which I have described in detail.

On microscopical study I find it to be identical with the other growth in structure, *i.e.*, merely granulation tissue. There were no parasites present in the stomach, and cultures made from the part failed to give anything save putrefactive forms. I also stained sections of the neoplasm for micro-organisms after the method of Gram, but failed to detect a single germ.

The cause of this ulcerative process is then still more in doubt than in the former case. In the other python there was nothing the matter with the gastric portion of its alimentary tract, but just beyond the pylorus there was an extensive ulcerative process, extending for a distance of eight or ten inches.

In the lower bowel I found a tapeworm of the genus dibothrium, and it evidently is an undescribed species. I have shown it to Dr. Leidy, who says he has never seen or read of one like it before.

The short space of time that intervenes between now and the date when this paper must be handed in will not allow of my inserting the results of the more thorough study I propose to give these specimens.

From the observations that I have already made upon them I am able to state the following points of interest: The gastric diverticulum in both snakes was at the pyloric end of the stomach, and contained a thick bronze-colored liquid of a disagreeable odor. I am also able to add to the different genera of parasites that have been found in ophidians the genus *dibothrium*.

## Figures and Tables

**Figure f1:**